# Silencing of *GATA3* defines a novel stem cell-like subgroup of ETP-ALL

**DOI:** 10.1186/s13045-016-0324-8

**Published:** 2016-09-22

**Authors:** L. Fransecky, M. Neumann, S. Heesch, C. Schlee, J. Ortiz-Tanchez, S. Heller, M. Mossner, S. Schwartz, L. H. Mochmann, K. Isaakidis, L. Bastian, U. R. Kees, T. Herold, K. Spiekermann, N. Gökbuget, C. D. Baldus

**Affiliations:** 1 Department of Hematology and Oncology, Charité, University Hospital Berlin, Campus Benjamin Franklin, Hindenburgdamm 30, 12203 Berlin, Germany; 2 Department of Hematology and Oncology, University Hospital Mannheim, Medical Faculty Mannheim of the University of Heidelberg, Mannheim, Germany; 3 Division of Children´s Leukaemia and Cancer, Telethon Kids Institute, University of Western Australia, Perth, Australia; 4 Department of Internal Medicine 3, University Hospital Grosshadern, Ludwig-Maximilians-Universität (LMU), Munich, Germany; 5 Department of Medicine II, Hematology/Oncology, Goethe University Hospital, Frankfurt/Main, Germany; 6 German Cancer Consortium (DKTK), Heidelberg, Germany

**Keywords:** *GATA3*, ETP-ALL, PER-117, Decitabine

## Abstract

**Background:**

*GATA3* is pivotal for the development of T lymphocytes. While its effects in later stages of T cell differentiation are well recognized, the role of *GATA3* in the generation of early T cell precursors (ETP) has only recently been explored. As aberrant *GATA3* mRNA expression has been linked to cancerogenesis, we investigated the role of *GATA3* in early T cell precursor acute lymphoblastic leukemia (ETP-ALL).

**Methods:**

We analyzed *GATA3* mRNA expression by RT-PCR (*n* = 182) in adult patients with T-ALL. Of these, we identified 70 of 182 patients with ETP-ALL by immunophenotyping. DNA methylation was assessed genome wide (Illumina Infinium® HumanMethylation450 BeadChip platform) in 12 patients and *GATA3*-specifically by pyrosequencing in 70 patients with ETP-ALL. The mutational landscape of ETP-ALL with respect to *GATA3* expression was investigated in 18 patients and validated by Sanger sequencing in 65 patients with ETP-ALL. Gene expression profiles (Affymetrix Human genome U133 Plus 2.0) of an independent cohort of adult T-ALL (*n* = 83) were used to identify ETP-ALL and investigate GATA3^low^ and GATA3^high^ expressing T-ALL patients. In addition, the ETP-ALL cell line PER-117 was investigated for cytotoxicity, apoptosis, *GATA3* mRNA expression, DNA methylation, and global gene expression before and after treatment with decitabine.

**Results:**

In our cohort of 70 ETP-ALL patients, 33 % (23/70) lacked *GATA3* expression and were thus defined as GATA3^low^. DNA methylation analysis revealed a high degree of *GATA3* CpG island methylation in GATA3^low^ compared with GATA3^high^ ETP-ALL patients (mean 46 vs. 21 %, *p* < 0.0001). Genome-wide expression profiling of GATA3^low^ ETP-ALL exhibited enrichment of myeloid/lymphoid progenitor (MLP) and granulocyte/monocyte progenitor (GMP) genes, while T cell-specific signatures were downregulated compared to GATA3^high^ ETP-ALL. Among others, *FLT3* expression was upregulated and mutational analyses demonstrated a high rate (79 %) of *FLT3* mutations. Hypomethylating agents induced reversal of *GATA3* silencing, and gene expression profiling revealed downregulation of hematopoietic stem cell genes and upregulation of T cell differentiation.

**Conclusions:**

We propose GATA3^low^ ETP-ALL as a novel stem cell-like leukemia with implications for the use of myeloid-derived therapies.

**Electronic supplementary material:**

The online version of this article (doi:10.1186/s13045-016-0324-8) contains supplementary material, which is available to authorized users.

## Background

*GATA3* is a transcription factor with a pivotal role in multiple developmental steps of T lymphopoiesis [1, 2], including the development of early T cell precursors (ETPs), a rare subpopulation of cells sharing characteristics with multipotent hematopoietic progenitors in the bone marrow [3]. ETPs are considered the most immature thymic cells with potential for complete T cell differentiation and retain plasticity for differentiation towards dendritic, NK, B, or myeloid cells [4]. In a murine model, *GATA3* was required for the development of ETPs, whereas totipotent hematopoietic stem cells (HSCs) remained unaffected by in vivo manipulation of *GATA3* expression levels. Indeed, in murine HSCs, *GATA3* was silenced by *DNMT3A*-dependent DNA hypermethylation [5]. By losing repressive epigenetic marks during T lymphopoiesis, *GATA3* functions as a key regulator of T cell differentiation through the interaction with a multitude of target genes that differ in a subpopulation specific manner [6]. For example, *GATA3* was reported to restrain Notch activity, repress NK-cell fate and upregulate T cell lineage genes to facilitate T cell differentiation [7].

Lack of *GATA3* has been linked to cancerogenesis, as absence of *GATA3* expression was associated with poor prognosis and undifferentiated tumors in breast cancer [8]. Moreover, several other cancers exhibited aberrant *GATA3* expression, including urothelial carcinoma [9], renal cell carcinoma [10], pancreatic cancer [11], cervical cancer [12], or Hodgkinʼs lymphoma [13]. In childhood B cell precursor acute lymphoblastic leukemia (BCP-ALL), specific germline variants of *GATA3* were associated with a higher incidence of BCP-ALL and a higher risk of relapse [14, 15].

Given *GATA3*’s prominent role in both cancerogenesis and T cell development, we investigated *GATA3* in ETP-ALL. ETP-ALL is a subtype of T-ALL characterized by a distinct gene expression profile (GEP) and a distinct immunophenotype with lack of CD1a and CD8, weak expression of CD5 and additional expression of more than 1 myeloid and/or stem cell marker [16]. ETP-ALL accounts for 11–15 % of cases with T-ALL [16–18] with similar distributions among pediatric and adult cohorts. We and others have characterized the mutational landscape of ETP-ALL with alterations in genes involved in cytokine and RAS signaling (e.g., *NRAS*, *KRAS*, *FLT3*, and *JAK1*), epigenetic regulation (e.g., *EZH2*, *DNMT3A*, and *SUZ12*), and hematopoietic development (e.g., *ETV6*, *RUNX1*, and *IKZF1*) [19, 20]. Notably, the incidence of activating *NOTCH1* mutations is considerably lower in ETP-ALL (15 %) when compared to T-ALL (higher than 50 %) [17, 21]. *GATA3* inactivating mutations were reported in 9 % of pediatric ETP-ALL patients predominantly affecting the DNA binding domain [19].

The prognostic relevance of ETP-ALL is controversially discussed. Comparing ETP-ALL with non-ETP-ALL, some reports indicate adverse prognosis in pediatric and adult patients with ETP-ALL with CR rates of 58–73 %, median event-free survival of 1.2 years, and 3-year overall survival of 30–60 % [16, 17, 22]. Other groups found similar outcome of ETP-ALL and non-ETP-ALL patients with 5-year overall survival rates of 67–93 and 77–92 %, respectively [23, 24].

Given the critical role that *GATA3* plays in early lymphoid development, we investigated *GATA3* in ETP-ALL, a stem cell-like leukemia blocked at the crossroads of lymphoid and myeloid differentiation. We hypothesized that aberrant *GATA3* expression would divert ETP-ALL from the lymphoid fate and determine a novel biological subgroup of ETP-ALL.

## Methods

### Patient samples

Additional file 1: Figure S1 provides an overview over the sample cohorts and subsequent experiments.

Gene expression data (Affymetrix HG-U133 Plus 2.0 or A + B) were available for adult T-ALL (*n* = 83; including 30 patients with ETP-ALL and 53 patients with non-ETP-ALL, defined by gene expression profiling [16], GEO accession number GSE78132), BCP-ALL (*n* = 81, GSE13204) [25], normal controls (NC; *n* = 24, GSE13204) [25], and acute myeloid leukemia (AML; *n* = 130) [26, 27]. The T-ALL subgroup included consecutive patients with newly diagnosed ALL studied between 1999 and 2005 at two reference laboratories [25, 28].

Based on immunophenotyping of diagnostic samples at the central diagnostic reference laboratory of the German Multicenter Study Group for Acute Lymphoblastic Leukemia (GMALL) in Berlin, Germany, we identified additional 70 ETP-ALL samples [17]. Sufficient RNA for *GATA3* mRNA expression analysis was available for all 70 samples, and sufficient genomic DNA (gDNA) for methylation assays was available for 69 samples of these adult ETP-ALL cases. As reference cohort, we used 112 non-ETP-ALL patients, of which 21 (19 %) had an immunophenotype of early T-ALL, 20 (18 %) of mature T-ALL, and 71 (63 %) of thymic T-ALL.

All patients, including the two independent cohorts of T-ALL, and normal controls gave written informed consent to participate in the study according to the Declaration of Helsinki. The studies were approved by the ethics board of the Johann Wolfgang von Goethe University, Frankfurt/Main, Germany.

### Nucleic acid preparation and molecular characterization

Pretreatment bone marrow and peripheral blood samples from patients were used for gDNA and total RNA extraction using TRIzol (Life Technologies, Grand Island, NY, USA) according to the manufacturer’s protocol with minor modifications. Complementary DNA (cDNA) was synthesized using 500 ng of total RNA and avian myeloblastosis virus reverse transcriptase (RT-AMV; Roche, Mannheim, Germany) in the presence of RNase inhibitor (RNasin; Roche, Mannheim, Germany).

**Fig. 1:**
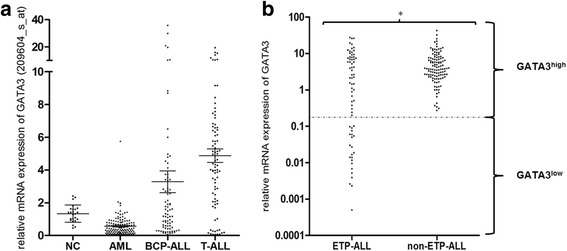
*GATA3* mRNA expression in patient samples. **a** Affymetrix-based mean differential expression of *GATA3* based on normalized expression values in normal controls (NC) and selected hematological disorders. *Horizontal lines* indicate mean *GATA3* expression $\pm$ s.e. Note the segmented *y*-axis. **b** RT-PCR-based analyses of *GATA3* mRNA expression relative to Jurkat on a logarithmic scale revealed lower *GATA3* expression in ETP-ALL (*n* = 70) than in non-ETP-ALL (*n* = 112) (4.82 vs. 6.29, *p* = 0.0005 indicated by *asterisk*). We identified a bimodal distribution of *GATA3* expression by K-means clustering with a cutoff at a relative expression of 0.2 (indicated by the *dotted line*). The GATA3^low^ cohort contained only cases with ETP-ALL (i.e., GATA3^low^ ETP-ALL)

Samples of patients with ETP-ALL (*n* = 70) and non-ETP-ALL (*n* = 112) were investigated by comparative multiplex real-time PCR (RT-PCR) for expression of *GATA3* (FWD: 5′-ACTACGGAAACTCGGTCAG-3′, REV: 5′-GTAGGGATCCATGAAGCAG-3′, Probe: 5′-CGGTGCAGAGGTACCCTCCG-3′) and *glucose*-*6*-*phosphate isomerase* (*GPI*) as a housekeeping gene. Relative *GATA3* expression values of ETP-ALL (*n* = 70) and non-ETP-ALL (*n* = 112) were normalized to *GATA3* expression in the human T-ALL cell line Jurkat. We identified a bimodal distribution of *GATA3* mRNA expression levels by K-means clustering and defined a cutoff at an expression level of 0.2 relative to Jurkat and defined all samples with *GATA3* expression below that cutoff as GATA3^low^ and samples with higher expression as GATA3^high^ (Fig. 1).

### Western blot

GATA3 protein levels were measured using standard western blotting techniques using the GATA3 antibody HG3-35 (Santa Cruz Biotechnology Inc., Dallas, TX, USA).

### Gene expression profiling

A GATA3-associated GEP was generated from data of 83 adult T-ALL patients (including 30 ETP-ALL and 53 non-ETP-ALL samples defined by hierarchical clustering using a list of genes reported as differentially expressed in pediatric ETP-ALL [16], GEO accession number GSE78132). For analysis, common probe sets between HG-U133 Plus 2.0 and HG-U133 A + B (Affymetrix, Santa Clara, CA, USA) were identified and quantile normalized. For ANOVA analysis, the type of chip was integrated as a random effect to take the batch effect into account. *GATA3* expression was calculated from signals obtained from probe sets 209602_s_at, 209603_at, and 209604_s_at, respectively. As for the RT-PCR expression levels, we identified a bimodal distribution of *GATA3*-normalized expression values and defined a cutoff at 8.2 on a logarithmic scale to categorize patients below that cutoff as GATA3^low^ and with higher expression as GATA3^high^. A *GATA3*-dependent GEP was generated by the comparison of the expression profiles from GATA3^low^ (*n* = 11) and GATA3^high^ (*n* = 72) samples. Lists of genes with at least 1.5-fold under- or overexpression comparing GATA3^low^ and GATA3^high^ were generated (Additional file 2: Table S1), and statistical significance was calculated by ANOVA with a FDR ≤0.05. Data analysis was carried out with Partek Genomic Suite v6.6 Software (Partek Inc., St. Louis, MO, USA).

Lists of up- and downregulated genes (Additional file 2: Table S1) were uploaded to the DAVID Bioinformatics server (http://david.abcc.ncifcrf.gov) to define enriched KEGG pathway annotations [29].

Additionally, GEP was performed on the Affymetrix® HG-U133 Plus 2.0 platform with ETP-ALL cell lines (PER-117, Loucy, in triplicates) and non-ETP-ALL cell lines (BE13, Molt4, Jurkat, RPMI8402; in duplicates) (GEO accession number GSE78166).

For gene set enrichment analysis (GSEA), *GATA3*-supervised GEPs were examined for enrichment of curated gene sets representing ETP-ALL [19], HSC [30], T cell differentiation [30], granulocyte/macrophage progenitors (GMP) [30], and myeloid/lymphoid progenitors (MLP) [31] comparing GATA3^low^ ETP-ALL (*n* = 11) and GATA3^high^ ETP-ALL (*n* = 19) cases. Additionally, we used decitabine-induced changes of GEP as a discriminator to analyze enrichment of these curated gene lists in PER-117 cells. Data analyses were carried out with the GSEA desktop application version 2.0.12 [32, 33] from the Broad Institute (http://www.broadinstitute.org/gsea).

### Methylation analysis

We assessed global DNA methylation analyses in 12 ETP-ALL and 14 BCP-ALL samples by the Illumina Infinium® HumanMethylation450 BeadChip platform. Hybridization was performed according to the manufacturer’s protocol. The signals generated for unmethylated and methylated cytosine nucleotides by single-nucleotide extension of locus-specific methylation probes were transformed into *β* values ranging from 0 to 1 (representing 0 to 100 %) for each of the 450,000 interrogated CpG residues. We assumed differential methylation, if more than three differentially methylated sites (DMS) with a *p* value <0.05 were present for each gene and the absolute difference of the corresponding *β* values $\left(\mathrm{\triangle}\beta \right)$ was greater than 0.17. Data analysis was carried out with Partek Genomic Suite v6.6 Software (Partek Inc., St. Louis, MO, USA).

Sufficient amounts of gDNA for bisulfite conversion was available for 69 ETP-ALL and 48 AML samples, which was carried out using the EpiTect Bisulfite Kit (QIAGEN, Hilden, Germany) according to the manufacturer’s instructions. For validation of the differentially methylated region of *GATA3* detected by global methylation analysis, primers were designed for amplification and pyrosequencing based on the bisulfite converted sequence of *GATA3* (genomic location: GRCh37: chr10:8097750-8098004) and used in the Pyrosequencing Assay Design Software v1.0 (Biotage, Uppsala, Sweden) for assay design. Amplification of a 255-bp sequence was carried out in all 69 bisulfite converted ETP-ALL samples using a 5′-GGAGGAGGTGGATGTGTTTTTTAAT-3′ forward and a 3′-AACCCCAATTTTTTTATAAATAAACCA-5′reverse biotinylated primer. Additionally, 13 representative samples of the non-ETP-ALL cohort were selected for analysis by pyrosequencing; 100 ng of bisulfite-converted gDNA was used per reaction with Taq-DNA-polymerase (Hot Start Mix S, peqlab, Erlangen, Germany). Samples were analyzed for specificity and correct size by 2 % agarose gel electrophoresis.

For pyrosequencing, a 5′-GTTACGGTGTAGAGGTATTTT-3′ sequencing primer was used. The percentage of CpG site methylation was calculated through the ratio of the relative content of thymine (i.e., unmethylated cytosine) and the relative content of cytosine (i.e., methylated cytosine) using the Pyro Q-CpG Software version 1.0.9 (Biotage, Uppsala, Sweden). Four of 12 CpG sites covered by the sequencing primer failed quality control due to the reference sequence pattern at the end of the amplicon. The remaining eight CpG sites were included to calculate the mean percentage of methylation for each sample.

### Cell culture and chemicals

The immature T-ALL cell line PER-117 [34] was grown in RPMI media with 10–20 % fetal bovine serum and cultured at 37 °C in a 5 % CO2 humidified chamber. PER-117 exhibited an immature phenotype resembling ETP-ALL (CD7^+^CD5^−^CD1a^−^cyCD3^+^CD33^+^TdT^−^CD10^−^CD34^−^CD117^−^), and gene expression profiling based on microarray analysis revealed an ETP-ALL phenotype (Additional file 3: Figure S2) including high expression of *GATA2*, *CEBPα*, or *NFE2* and low expression of *LEF1* and *GATA3* (GATA3^low^ ETP-ALL).

Additionally, the ETP-ALL cell line Loucy (with high *GATA3* expression, GATA3^high^ ETP-ALL) and the non-ETP-ALL cell lines, Jurkat, Molt4, BE13, and RPMI8402 were obtained from the German Resource Center for Biological Material, DSMZ (Braunschweig, Germany) and previously characterized on a molecular level [35]. 5-Azacytidine and 5-aza-deoxycytidine were purchased from Sigma-Aldrich (St. Louis, MO, USA).

### Cell proliferation assay

Cell proliferation was measured with the WST-1 reagent according to the manufacturer’s instructions (Roche Diagnostics GmbH, Germany). Cell lines were treated with various concentrations of 5-azacytidine (Sigma-Aldrich, St. Louis, USA) and 5-aza-deoxycytidine (Sigma-Aldrich, St. Louis, USA), and absorbance was measured after 48, 72, and 96 h by optical density absorption analyses at a wavelength of 450 nm using an ELISA multiplate reader.

### Apoptosis assay

Apoptosis was measured using Annexin V Apoptosis Detection Kit (BD Pharmingen, Heidelberg, Germany). Cells were labeled with Annexin V and 7-amino-actinomycin D (7-AAD) after treatment with 5-azacytidine and 5-aza-deoxycytidine. Analyses were performed by FACS Calibur (Becton-Dickinson) to determine the percentage of apoptotic cells from combined 7-AAD incorporation and Annexin V binding.

### Statistical analysis

The statistical difference of gene expression between two independent groups was tested by the non-parametric Mann-Whitney *U* test. For non-parametric correlation of mRNA expression and DNA methylation, Spearman’s rank correlation coefficient was calculated. Fisher’s exact test was used to test for the association between two kinds of classifications (e.g., 2 × 2 contingency table).

For all tests, a *p* value <0.05 (two-sided) was considered to indicate a significant difference. All calculations were performed using the SPSS software version 19 (SPSS Inc., Chicago, IL, USA), GraphPad Prism® software version 5 (GraphPad Software Inc., La Jolla, CA, USA), and Partek Genomic Suite v6.6 Software (Partek Inc., St. Louis, MO, USA).

## Results

### Lack of GATA3 expression in ETP-ALL

We first assessed *GATA3* mRNA expression by microarray analysis and found that mean expression of *GATA3* was higher in T-ALL (4.88 ± 0.41, mean ± s.e., *n* = 83) than in other cohorts (NC 1.33 ± 0.11, *n* = 24; AML 0.57 ± 0.05, *n* = 130; and BCP-ALL 3.28 ± 0.66, *n* = 81; all values are mean ± s.e., *p* < 0.001) (Fig. 1a).

To further explore *GATA3* expression in T-ALL, we analyzed *GATA3* mRNA expression by quantitative RT-PCR in larger cohorts of ETP-ALL (*n* = 70) and non-ETP-ALL (*n* = 112). The mean relative expression of *GATA3* was lower in ETP-ALL than in non-ETP-ALL (4.82 ± 0.78 vs. 6.29 ± 0.60, mean ± s.e., *p* = 0.0005). Interestingly, we found a bimodal distribution of *GATA3* expression with one third of ETP-ALL patients lacking *GATA3* expression (23/70, 33 %, GATA3^low^ ETP-ALL). In contrast, none of 112 non-ETP-ALL samples lacked *GATA3* expression, which consisted of 71 thymic, 21 early, and 20 mature T-ALL patient samples (Fig. 1b). In agreement with this, the non-ETP-ALL cell lines Molt4, Jurkat, RPMI8402, and BE13 all expressed *GATA3*, while PER-117 [34], a cell line with an ETP-ALL immunophenotype and GEP (Additional file 3: Figure S2) lacked *GATA3* expression. Western blotting revealed that differential *GATA3* mRNA expression translated into differential protein expression levels (Additional file 4: Figure S3).

### GATA3 silencing is mediated by aberrant DNA methylation

**Fig. 2:**
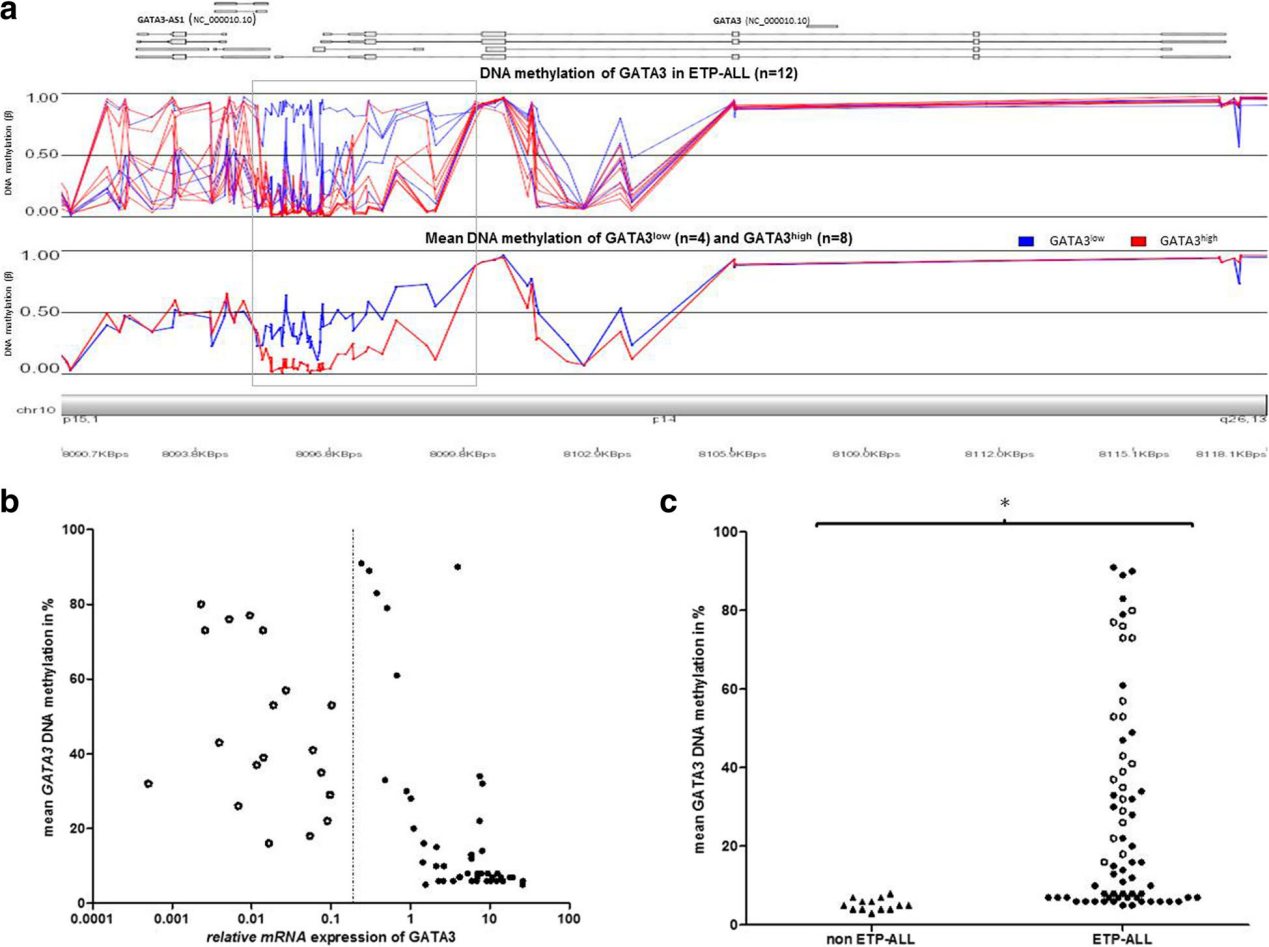
*GATA3* silencing is regulated by DNA methylation in ETP-ALL. **a** 10p15 chromosome plot depicting DNA methylation of 12 ETP-ALL patient samples as assessed by Illumina Infinium® HumanMethylation450 BeadChip. *β* values representing DNA methylation for each patient (*upper panel*) and mean DNA methylation (*lower panel*) of GATA3^low^ ETP-ALL (*n* = 4, *blue*) and GATA3^high^ ETP-ALL (*n* = 8, *red*). Comparing GATA3^low^ ETP-ALL (*n* = 4, *blue*) with GATA3^high^ ETP-ALL (*n* = 8, *red*), 35 differentially methylated sites were located within a 6-kb segment of *GATA3* (indicated by the *gray box*), including the CpGs that were analyzed by pyrosequencing in a larger cohort of patients. **b** *GATA3* DNA methylation as assessed by pyrosequencing (of CpGs within the *gray box* in **a**) was negatively correlated to *GATA3* mRNA expression in ETP-ALL (*n* = 64, *r* = −0.73, *p* < 0.0001). The *dotted line* indicates the cutoff to distinguish GATA3^low^ (*empty dots*) and GATA3^high^ (*solid dots*) samples. **c** Pyrosequencing revealed higher *GATA3* DNA methylation in ETP-ALL (*n* = 69) than in non-ETP-ALL (*n* = 13) (28 vs. 5 %, *p* < 0.0001 indicated by *asterisk*). *Empty* and *solid dots* indicate GATA3^low^ and GATA3^high^ ETP-ALL, while *triangles* indicate non-ETP-ALL

To explore the regulation of *GATA3* expression, we investigated global DNA methylation on the Illumina HumanMethylation 450 k platform in 12 ETP-ALL samples (Fig. 2), which were selected according to *GATA3* mRNA expression (GATA3^low^ vs. GATA3^high^) and mutational status of *DNMT3A*. The genomic locus of *GATA3* (NC_000010.10) was represented by 72 CpG sites.

Four of 12 ETP-ALL patients were defined as GATA3^low^ (mean *GATA3* expression ± s.e.: 0.08 ± 0.05), while the remaining eight patients were GATA3^high^ (mean *GATA3* expression ± s.e.: 6.4 ± 1.5). *GATA3* DNA methylation of all 72 *GATA3* CpG sites was higher in GATA3^low^ ETP-ALL compared to GATA3^high^ ETP-ALL (mean 45 vs. 23 %, *p* < 0.0001). We detected 35 of 72 *GATA3* CpG sites with a significantly higher degree of methylation in GATA3^low^ ETP-ALL compared to GATA3^high^ ETP-ALL (mean 46 vs. 19 %, *p* < 0.0001). All 35 DMS clustered in a 6-kb region (genomic location: GRCh37: chr10:8095478-8101513; indicated by the grey box in Fig. 2a), which mapped to a *GATA3* CpG island (GRCh37: chr10:8091375-8098329) that was previously reported to be differentially methylated in cancer [10, 36].

Comparing *DNMT3A*-mutated (*n* = 6) and *DNMT3A* wild-type (*n* = 6) ETP-ALL, we found lower *GATA3* methylation in *DNMT3A*-mutated versus *DNMT3A* wild-type samples (16 vs. 35 %, *p* < 0.0001) at the *GATA3* CpG island (GRCh37: chr10:8091375-8098329), but *GATA3* expression, as determined by RT-PCR, was not different between the *DNMT3A* mutated (*n* = 6) and wild-type (*n* = 6) ETP-ALL cases (4.4 vs. 3.8, *p* = 0.84). Notably, all 16 DMS within the *GATA3* CpG island clustered in a 3.3-kb region (GRCh37: chr10:8092037-8095363) just upstream of the 6-kb region that correlated to *GATA3* gene mRNA expression, but remarkably without overlap.

To validate these findings in a larger sample set, DNA methylation was analyzed by pyrosequencing in 69 ETP-ALL samples; 11 of 69 samples were also investigated by the Illumina Human Methylation assay. Capturing a segment of the *GATA3* CpG island (GRCh37: chr10:8097750-8098004) (Additional file 5: Figure S4), we assessed 64 samples of which both *GATA3* mRNA expression and *GATA3* DNA methylation were available. We confirmed a high degree of concordance between pyrosequencing and the Illumina Human Methylation assay in samples analyzed in parallel on both platforms (*n* = 11, *R*^2^ = .94). By pyrosequencing, we confirmed a higher degree of DNA methylation in GATA3^low^ ETP-ALL (*n* = 19) compared to GATA3^high^ ETP-ALL (*n* = 45) (mean 46 vs. 21 %, *p* < 0.0001). *GATA3* expression and DNA methylation were inversely correlated (*r* = −0.73, *p* < 0.0001) (Fig. 2b). When we compared ETP-ALL to non-ETP-ALL, DNA methylation was lower in non-ETP-ALL (5 %, range 3–8 %, *n* = 13) than in ETP-ALL (28 %, range 5–91 %, *n* = 69; *p* < 0.0001) (Fig. 2c).

### GATA3^low^ ETP-ALL is associated with FLT3 mutations

Our group previously assessed the mutational landscape of ETP-ALL by whole exome sequencing [20] and targeted NGS re-sequencing [37]. Within this cohort of ETP-ALL, we have investigated the mutational pattern with respect to *GATA3* expression (Additional file 6: Table S2). In contrast to pediatric cohorts, we found no *GATA3* mutations in this cohort, including a screen for hotspot mutations of exon 4 in an additional expansion cohort of 70 samples of adult ETP-ALL.

Comparing GATA3^low^ and GATA3^high^ ETP-ALL, we found *FLT3* mutations (including both internal tandem duplications and mutations in the tyrosine kinase domain) in 79 % of GATA3^low^ ETP-ALL (15/19), while only 15 % of GATA3^high^ ETP-ALL (7/46) were *FLT3* mutated (*p* < 0.001). Comparing *FLT3*^+^- and *FLT3*^−^-ETP-ALL, we found lower *GATA3* expression (0.5 vs. 7.0, *p* < 0.0001) and higher *GATA3* methylation as detected by pyrosequencing (51 vs. 8 %, *p* < 0.0001) in *FLT3*^+^-ETP-ALL. *NOTCH1* mutations were generally infrequent in our cohort of ETP-ALL (11/65, 17 %) and less frequent still in GATA3^low^ ETP-ALL than in GATA3^high^ ETP-ALL (2/19, 11 % vs. 9/46, 20 %). Notably, we detected mutations in genes of the PRC2 complex in only a small number of adult ETP-ALL patients (5/70, 7 %) with marginal differences between GATA3^low^ and GATA3^high^ ETP-ALL cases (2/23, 9 vs. 3/47, 6 %). *DNMT3A*, *EZH2*, *SUZ12*, and *EP300* mutations were similarly distributed among GATA3^low^ and GATA3^high^ samples as well as all other investigated genes.

### Distinct transcriptional program of GATA3^low^ ETP-ALL

**Fig. 3:**
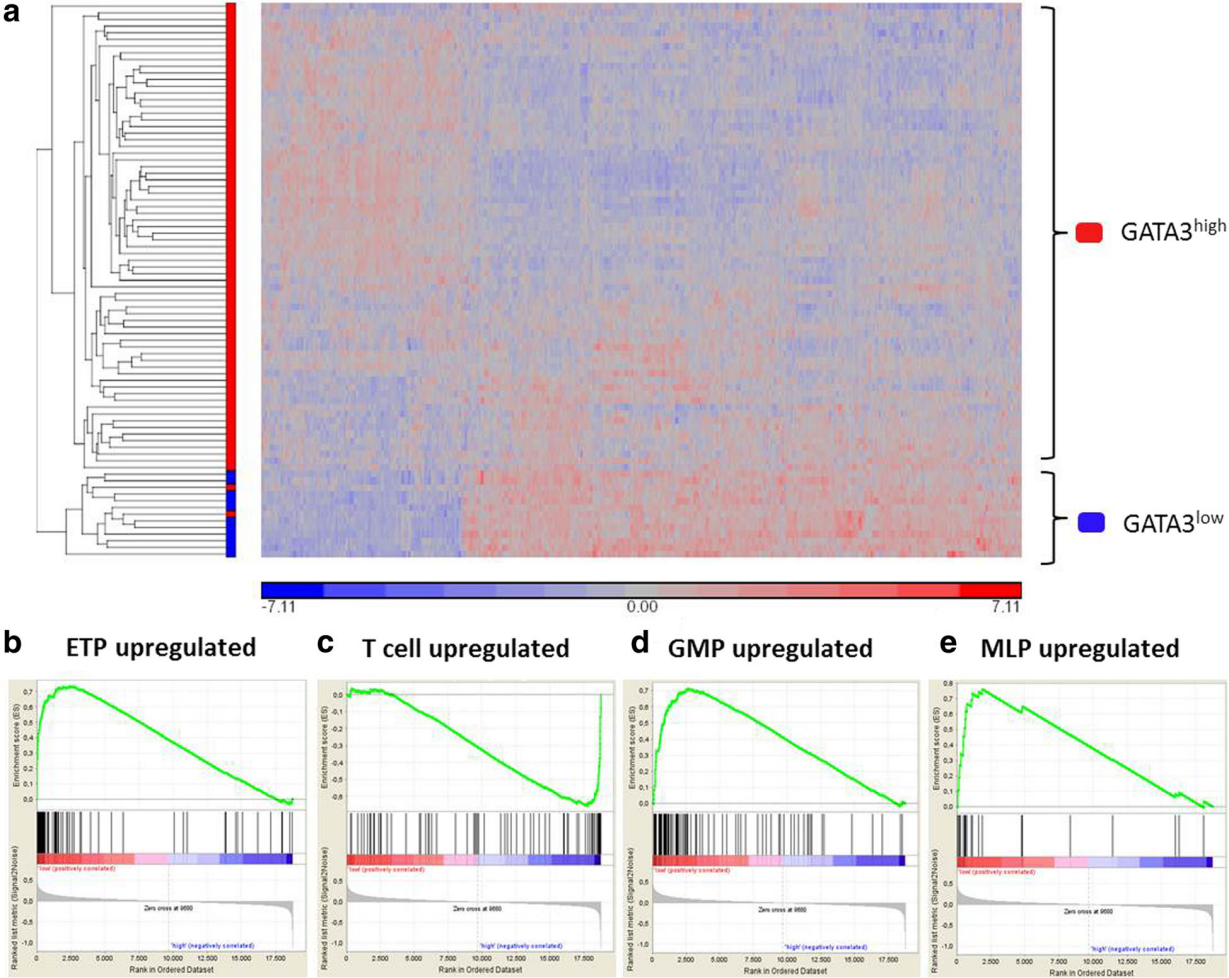
GATA3^low^ ETP-ALL exhibits a distinct transcriptional program. **a** Heat map of 1.5-fold DEG comparing GATA3^low^ (*n* = 11) with GATA3^high^ (*n* = 72) in patients with T-ALL (*n* = 83), including 30 patients with ETP-ALL and 53 patients with non-ETP-ALL. **b**–**e** GSEA restricted to ETP-ALL (*n* = 30) comparing GATA3^low^ (*n* = 11) and GATA3^high^ ETP-ALL (*n* = 19) revealed enrichment of ETP-ALL genes (NES = 1.5, *p* < 0.01) (**b**), depletion of T cell differentiation genes (NES = 1.6, *p* < 0.008) (**c**), enrichment of GMP-based genes (NES = 1.5, *p* = 0.06) (**d**), and enrichment of MLP-based genes (NES = 1.5, *p* = 0.04) in GATA3^low^ ETP-ALL (**e**)

To explore differences of the transcriptional program, microarray expression data of 83 T-ALL patients were available; 11 of 83 patients were defined as GATA3^low^, while the remaining 72 patients were classified as GATA3^high^. Including probe sets with at least 1.5-fold overexpression, we detected 1435 differentially expressed probes sets in GATA3^low^ compared to GATA3^high^ T-ALL cases (Additional file 2: Table S1). Hierarchical clustering with this gene list revealed a GATA3^low^-derived gene expression signature (Fig. 3a). Importantly, this GATA3^low^ GEP identified all but one case of ETP-ALL in an independent cohort of pediatric T-ALL (Additional file 7: Figure S5) [16]. Annotation of the top 267 DEG (i.e., genes with fold change ≥3×) using the KEGG pathway database demonstrated significant enrichment of upregulated genes associated to cancer and, notably, AML, while genes associated to T cell signaling were downregulated in GATA3^low^ samples (Additional file 8: Table S3).

Next, we intended to dissect molecular differences by comparing GATA3^low^ and GATA3^high^ in cases with ETP-ALL only. In a cohort of 30 adult patients with ETP-ALL, we identified 11 patient samples as GATA3^low^ and 19 as GATA3^high^ (Additional file 9: Figure S6). Applying GSEA, we found significant enrichment of ETP-ALL-associated genes (NES = 1.51, *p* = 0.01, FDR = 0.05) (Fig. 3b) and depletion of genes involved in T cell differentiation (NES = −1.6, *p* = 0.008, FDR = 0.01) in GATA3^low^ ETP-ALL cases (Fig. 3c). Moreover, we also found enrichment for GMP-based genes (NES = 1.5, *p* = 0.06, FDR = 0.1, Fig. 3d) and MLP-based genes (NES = 1.5, *p* = 0.04, FDR = 0.14, Fig. 3e) in the GATA3^low^ group.

### Decitabine restores GATA3 expression in PER-117 cells

Given the high degree of *GATA3* DNA methylation, we studied whether hypomethylating agents (HMA) could convert methylation-induced *GATA3* silencing. We used PER-117 as a model for GATA3^low^ ETP-ALL with high *GATA3* DNA methylation (mean DNA methylation 92 % $\pm$ 1 %), low *GATA3* mRNA expression (relative expression to Jurkat 0.002), and an ETP-like immunophenotype and GEP (Additional file 3: Figure S2). We evaluated *GATA3* DNA methylation by pyrosequencing and *GATA3* expression by RT-PCR after treatment with decitabine.

**Fig. 4:**
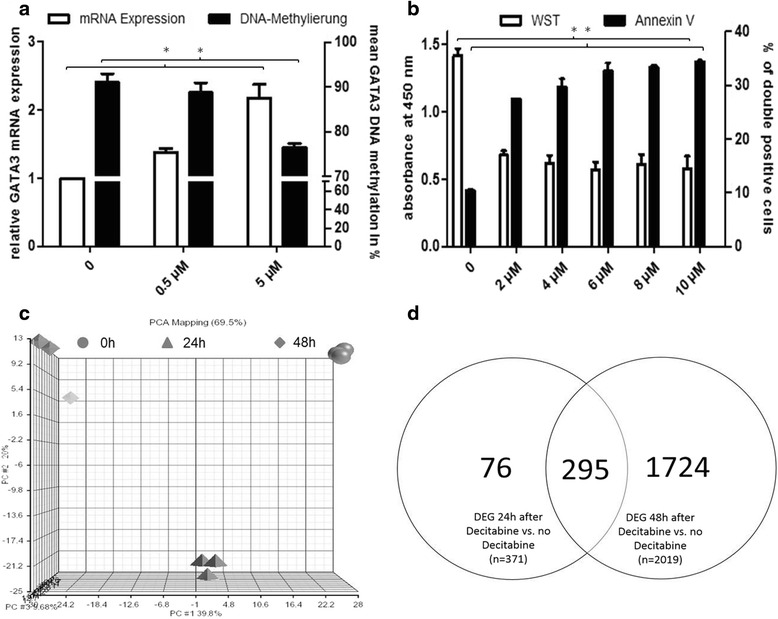
Decitabine reverses *GATA3* silencing in PER-117 cells, an in vitro model for GATA3^low^ ETP-ALL. **a** Treatment with decitabine (5 μM) increased *GATA3* mRNA expression (*n* = 4, *p* < 0.001 indicated by the *asterisk*, all values are mean ± s.d.) and decreased *GATA3* DNA methylation after 48 h (*n* = 4, *p* < 0.05 indicated by the *asterisk*, all values are mean ± s.d.) as detected by pyrosequencing. Note the two segments of the right *y*-axis for improved visualization. **b** Treatment with decitabine impaired proliferation as detected by WST assay with an IC50 of 4 μM and induced apoptosis as detected by flow cytometry of incorporation of 7-AAD and Annexin V binding after 48 h (*n* ≥ 5, *p* < 0.05 indicated by the *asterisk*, all values are mean ± s.d). **c** Principal component analysis of global gene expression profiles of PER-117 cells before and after treatment with decitabine (5 μM). **d** Venn diagram indicating the number of differentially expressed probe sets in PER-117 cells after 24 and 48 h and at both time points compared with no treatment

Treatment of PER-117 with decitabine (48 h, 5 μM) increased *GATA3* mRNA expression by 2.2-fold (*n* = 4, *p* < 0.001) while lowering *GATA3* DNA methylation from 91 to 78 % (*n* = 4, *p* < 0.05) (Fig. 4a). In contrast, another ETP-ALL cell line, Loucy, exhibited higher *GATA3* expression (GATA3^high^ ETP-ALL) than PER-117 and treatment with decitabine failed to induce *GATA3* expression. In PER-117, decitabine induced 50 % growth inhibition at a concentration (IC_50_) of 4 μM (*n* = 9, *p* < 0.05) and enhanced apoptosis at the IC_50_ from 10 to 29 % after 48 h (*n* = 5, *p* < 0.05) (Fig. 4b).

We analyzed global gene expression of PER-117 cells by Affymetrix microarrays before and after treatment with decitabine at a final concentration of 5 μM at three time points (0, 24, and 48 h). At both 24 and 48 h after decitabine treatment, we detected significant changes in global gene expression compared to untreated cells (Fig. 4c) with 2019 differentially expressed probe sets (fold change of ≥1.5 and FDR <0.05) after 48 h of exposure to decitabine (Fig. 4d). Principal component analysis revealed differential changes of global gene expression after 24 and 48 h: GEP changes represented by the first principal component expanded up until 48 h, while GEP changes subsumed by the second and third principal components were nearly completely reversible after 48 h (Fig. 4c).

Pathway analysis of all DEG after 48 h of decitabine treatment identified significant upregulation of p53 signaling genes (e.g., *FAS*, *CDKN1a*, or *MDM2*), while genes involved in cell cycling (e.g., *CDK6*, *RB1*, *GSK3B*, or *E2f5*) were downregulated upon treatment with decitabine. Moreover, we found downregulation of cancer-associated genes such as *BCL2*, *FLT3LG*, or *PIK3r5* in cells treated with decitabine (Additional file 10: Table S4). Importantly, *GATA3* ranked among the top upregulated genes (fold change of 2.2, *p* < 0.0001) confirming doubled *GATA3* mRNA expression levels determined by RT-PCR.

To further characterize the transcriptional changes upon decitabine treatment, we performed GSEA comparing untreated with treated PER-117 cells. In cells treated with decitabine, we found downregulation of HSC genes (NES = 1.28, *p* < 0.001, FDR = 0.16) and, in line with increased *GATA3* expression, upregulation of T cell differentiation (NES = 1.16, *p* = 0.06, FDR = 0.22).

## Discussion

Here, we discovered a novel, molecularly distinct subgroup of T-ALL patients lacking *GATA3* expression (GATA3^low^). All GATA3^low^ T-ALL patients exhibited an immunophenotype of ETP-ALL, while GATA3^high^ T-ALL patients were of thymic, early, or mature subtypes. The subgroup of GATA3^low^ ETP-ALL is molecularly and clinically relevant as it lacks T lineage commitment in favor of a sustained myeloid gene expression signaling and a high rate of *FLT3* mutations.

Clustering analysis revealed a third of our cohort’s ETP-ALL samples to be GATA3^low^. To study mechanisms of silenced *GATA3* mRNA expression, we investigated DNA methylation. We identified a CpG island of *GATA3* with consistently higher *GATA3* DNA methylation in GATA3^low^ ETP-ALL compared to GATA3^high^ ETP-ALL including more than 30 DMS. This *GATA3* CpG island was differentially methylated in renal cell carcinoma [10] and thyroid adenocarcinoma. In fact, cg01255894, a hypermethylated CpG site in ETP-ALL, was among the top 25 methylation probes that were most negatively correlated with gene expression [36]. Notably, *GATA3* DNA hypermethylation was absent in non-ETP-ALL indicating that *GATA3* silencing was a distinct mechanism in ETP-ALL. It is tempting to relate this finding to reports of murine *DNMT3A*-deficient mice, where *GATA3* silencing was associated with *DNMT3A*-dependent DNA hypermethylation in HSC [5]. Indeed, when we compared *DNMT3A* mutated and *DNMT3A* wild-type ETP-ALL, we found lower *GATA3* DNA methylation in samples with mutated *DNMT3A*, but *GATA3* mRNA expression was not different between *DNMT3A* wild-type and mutated ETP-ALL. Thus, *DNMT3A* contributes to *GATA3* DNA methylation; however, redundant mechanisms are likely required for *GATA3* silencing in GATA3^low^ ETP-ALL. Importantly, hypermethylation of *GATA3* was found only in the subset of GATA3^low^ ETP-ALL, but not in other leukemic subtypes such as typical T-ALL or BCP-ALL. Notably, in 49 samples from patients with AML, *GATA3* expression was similarly low as in GATA3^low^ ETP-ALL (mean 0.2 vs. 0.03), but DNA hypermethylation was absent in AML (17 vs. 46 %). Thus, GATA3^low^ ETP-ALL may reflect the transformed precursor stage of yet non-committed ETP that physiologically harbor *GATA3* DNA hypermethylation.

In order to explore the cell of origin of GATA3^low^ ETP-ALL, we identified a GATA3^low^-specific GEP in a cohort of T-ALL, including ETP-ALL and non-ETP-ALL patient samples. GATA3^low^ and GATA3^high^ samples generated distinct gene expression clusters in a supervised analysis. The biological significance of this observation was underscored when we validated our GATA3^low^ signature by identifying cases with ETP-ALL in an independent cohort of pediatric patients with T-ALL [16] by unsupervised hierarchical clustering. Moreover, pathway annotation of DEG indicated upregulation of myeloid genes and downregulation of T cell differentiation. Perhaps unsurprisingly, we found depletion of T cell signaling and enrichment of myeloid signaling when we performed GSEA comparing GATA3^low^ ETP-ALL with T-ALL. By restricting the analysis to ETP-ALL only, we confirmed enrichment of GMP and MLP signatures and depletion of T cell differentiation in GATA3^low^ samples compared to GATA3^high^ ETP-ALL, which pointed at a specific molecular bracket within ETP-ALL. The specificity of *GATA3* in this regard was further underscored when we analyzed other relevant transcription factors involved in T cell differentiation. Other transcription factors, such as *MEF2C*, *PU.1*, *BCL11B*, *LMO1*-*3*, *HOXA1*, *TCF*-*1*, or *LYL1* failed to identify subsets with meaningful gene set enrichment in neither “typical” T-ALL nor ETP-ALL. Only the transcription factor *LEF1* segregated cases into subgroups with similar gene set enrichment patterns as *GATA3* subgroups, albeit with significant overlap of GATA3^low^/LEF1^low^ cases. *LEF1* is an important effector of *WNT* signaling and, like *GATA3*, known to be essential for early stages of T cell development. In T cell malignancy, *LEF1* was implicated in transforming T cells in the absence of *TCF1* [38].

The observation of a myeloid gene expression signature was further supported by the high frequency of *FLT3* mutations in GATA3^low^ ETP-ALL. It is important to note that neither of the investigated cases fulfilled the diagnostic criteria for leukemia of ambiguous lineage or acute myeloid leukemia. Therefore, these findings point to T-lymphoblastic precursors with multilineage potential as cells of origin of GATA3^low^ ETP-ALL. Indeed, enrichment of ETP-ALL genes in GATA3^low^ compared with GATA3^high^ ETP-ALL reinforced this assumption as ETP-ALL by itself is characterized by upregulation of stem cell genes and myeloid-derived gene expression [19].

Ultimately, the significance of GATA3^low^ ETP-ALL as a subgroup of ETP-ALL will depend on the implementation of distinct therapeutic interventions. In our ETP-ALL cohort, we found no significant outcome differences comparing GATA3^low^ and GATA3^high^ ETP-ALL (1-year OS 75 vs. 79 %) in a retrospective analysis of 52 patients. In general, the clinical outcome of ETP-ALL remains controversial, as reports of adverse risk in pediatric and adult ETP-ALL [16, 22, 39] have been challenged by reports indicating no outcome differences between ETP-ALL and non-ETP-ALL patient cohorts [23, 24]. This controversy might be in part due to the definition of ETP-ALL by GEP or flow cytometry as well as differences in treatment intensities, especially MRD-directed approaches to treatment intensification [16, 19, 23, 24, 40].

In any case, the mutational and transcriptional profile of GATA3^low^ ETP-ALL provides rationale for implementing targeted therapies in patients with failure of lymphoid-directed therapies. Low incidence of *NOTCH1* mutations in GATA3^low^ ETP-ALL will likely render NOTCH-targeted therapies (e.g., γ-secretase inhibitors) ineffective. On the other hand, *FLT3* mutations were detected in more than 75 % of GATA3^low^ ETP-ALL and our group has previously shown in vitro efficacy of *FLT3* inhibitors in human T-ALL cell lines [17]. Importantly, *GATA3* DNA hypermethylation implicates epigenetic therapies in GATA3^low^ ETP-ALL, such as decitabine, a hypomethylating agent approved for the treatment of myelodysplastic syndrome, chronic myelomonocytic leukemia, and AML. Our data demonstrate that decitabine induced apoptosis in PER-117 cells while lowering *GATA3* DNA methylation. Subsequent induction of *GATA3* expression may function as a surrogate of T cell differentiation which we also observed in PER-117 cells upon decitabine treatment. This is in line with murine breast cancer, where lack of *GATA3* is associated with undifferentiated tumors [8]. The IC_50_ of decitabine in our experiments was comparable to AML cell lines [41, 42], and current dosing of decitabine in AML results in a similar range of steady state plasma levels [41]. Although further experiments are necessary to evaluate in vivo efficacy of HMA in T-ALL, it is likely that similar doses of decitabine will be required in T-ALL and AML. It is important to note that forced *GATA3* overexpression alone failed to induce relevant changes in proliferation, apoptosis, or differentiation in PER-117 cells, which we attributed to *GATA3* dose sensitivity. Instead, subtle changes of *GATA3* expression are needed to divert aberrant DNA hypermethylation towards an equilibrium of optimum methylation in T lymphoblasts [43], for which *GATA3* induction upon treatment with decitabine serves as yet another example.

## Conclusions

*GATA3* silencing occurs in about one third of adult ETP-ALL patients and is associated with *GATA3* DNA hypermethylation. Lack of *GATA3* engages a transcriptional program that is characterized by enrichment of myeloid signatures and loss of T cell differentiation against a background of both T-ALL and ETP-ALL. We propose a novel stem cell-like leukemia termed GATA3^low^ ETP-ALL with a high frequency of *FLT3* mutations as a distinct molecular entity with sensitivity to hypomethylating agents.

## Additional files

Additional file 1: Figure S1. Flowchart depicting the patient sample cohorts. Strategy to define ETP-ALL is provided as well as platforms for subsequent analyses. (JPG 93 kb) Additional file 2: Table S1. List of DEG comparing patients with low and high *GATA3* mRNA expression. Microarray analyses of diagnostic bone marrow samples of 83 adult patients with T-ALL were used to generate a list of DEG comparing GATA3^low^ and GATA3^high^. Only probe sets with at least 1.5-fold up- or downregulation with respect to *GATA3* expression and a FDR <0.05 were included. (XLSX 102 kb) Additional file 3: Figure S2. PER-117 and Loucy are ETP-ALL cell lines. Hierarchical clustering of ETP-ALL and non-ETP-ALL cell lines, using a set of genes differentially expressed in pediatric patients with ETP-ALL compared with non-ETP-ALL [20]. (PNG 63 kb) Additional file 4: Figure S3.
*GATA3* expression in human leukemia cell lines. (A) RT-PCR-based analysis of *GATA3* mRNA expression relative to Jurkat indicates differential *GATA3* mRNA expression. (B) Western blot analysis revealed differential GATA3 protein expression (predicted molecular weight of 47 kDa). (JPG 35 kb) Additional file 5: Figure S4. Pyrograms showing *GATA3* DNA methylation in ETP-ALL in eight consecutive CpG sites across 64 bases. The sequence of the *GATA3* amplicon is indicated at the top, while the sequence at the bottom of each panel indicates the dispensation order. The grey boxes highlight peaks resulting from sequential dispensations of C and T from which DNA methylation was calculated (blue boxes). (A) Representative ETP-ALL sample exhibiting high *GATA3* DNA methylation. (B) Representative ETP-ALL sample exhibiting low *GATA3* DNA methylation. (PNG 226 kb) Additional file 6: Table S2. Next generation sequencing comparing the mutational pattern of GATA3^low^ (*n* = 5) and GATA3^high^ ETP-ALL (*n* = 13). Sanger Sequencing was performed for selected genes in an extended cohort (*n* = 70) confirming a high rate of *FLT3* mutations in GATA3^low^ ETP-ALL. (PNG 47 kb) Additional file 7: Figure S5. The GATA3^low^ gene expression profile identified cases with ETP-ALL in an independent cohort of T-ALL. Unsupervised hierarchical cluster analysis of diagnostic bone marrow samples from 55 pediatric patients with T-ALL, using a set of genes defined by low *GATA3* expression in adult T-ALL. Classification of ETP-ALL and non-ETP-ALL was performed according to the ETP gene expression signature published along with this dataset. (PNG 484 kb) Additional file 8: Table S3. KEGG pathway annotation of DEG in GATA3^low^ versus GATA3^high^ T-ALL patients. Lists of threefold over- and underexpressed genes of GATA3^low^ patients were uploaded to DAVID Bioinformatics Resources v6.7. Listed are selected KEGG annotated pathway elements significantly enriched in GATA3^low^ up- or downregulated DEGs. (PNG 34 kb) Additional file 9: Figure S6. Validation of the GATA3^low^ ETP-ALL cluster in adult patients with T-ALL using the ETP signature derived from pediatric T-ALL. Unsupervised hierarchical cluster analysis of diagnostic bone marrow samples from 83 adult patients with T-ALL, using a set of genes defined in pediatric T-ALL [20]. Blue = GATA3^low^ ETP-ALL (*n* = 11), red = GATA3^high^ (*n* = 72). Patients assigned to ETP-ALL and “typical” T-ALL are indicated. (PNG 748 kb) Additional file 10: Table S4. KEGG pathway annotation of DEG in PER-117 cells before versus after treatment with decitabine. Lists of 1.5-fold over- and underexpressed genes of cells treated with decitabine (5 μM) were uploaded to DAVID Bioinformatics Resources v6.7. Listed are selected KEGG annotated pathway elements significantly enriched in cells treated with decitabine. (PNG 61 kb)
