# Supplementary figures and images for: Silencing of GATA3 defines a novel stem cell-like subgroup of ETP-ALL
Source: J Hematol Oncol. 2016 Sep 22;9:95. doi: 10.1186/s13045-016-0324-8 (PMC5034449; doi:10.1186/s13045-016-0324-8)

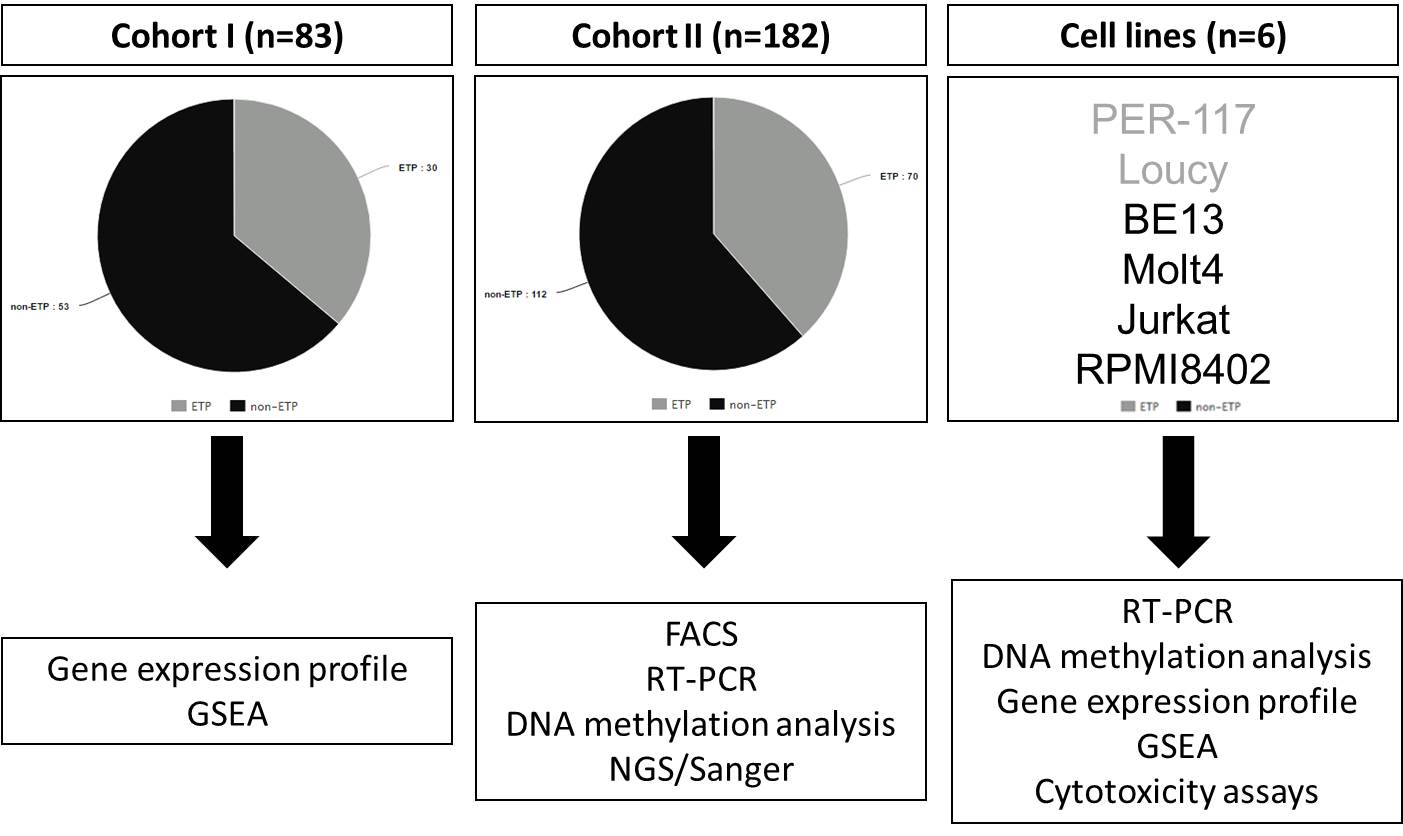

Supplement: Additional file 1: Figure S1. — Flowchart depicting the patient sample cohorts. Strategy to define ETP-ALL is provided as well as platforms for subsequent analyses. (JPG 93 kb) [file 13045_2016_324_MOESM1_ESM.jpg]

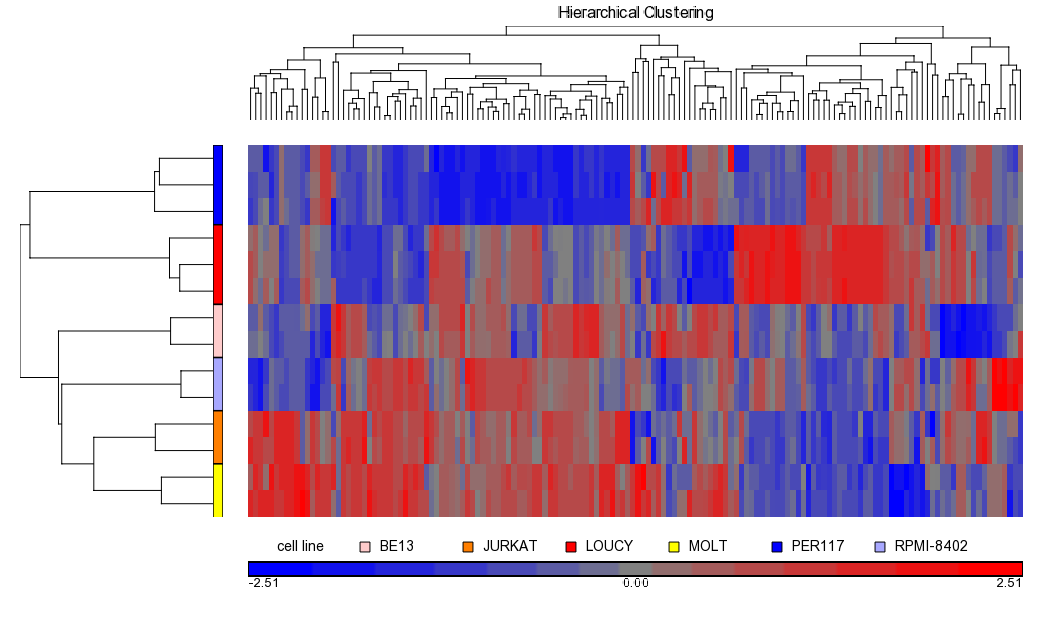

Supplement: Additional file 3: Figure S2. — PER-117 and Loucy are ETP-ALL cell lines. Hierarchical clustering of ETP-ALL and non-ETP-ALL cell lines, using a set of genes differentially expressed in pediatric patients with ETP-ALL compared with non-ETP-ALL [20]. (PNG 63 kb) [file 13045_2016_324_MOESM3_ESM.png]

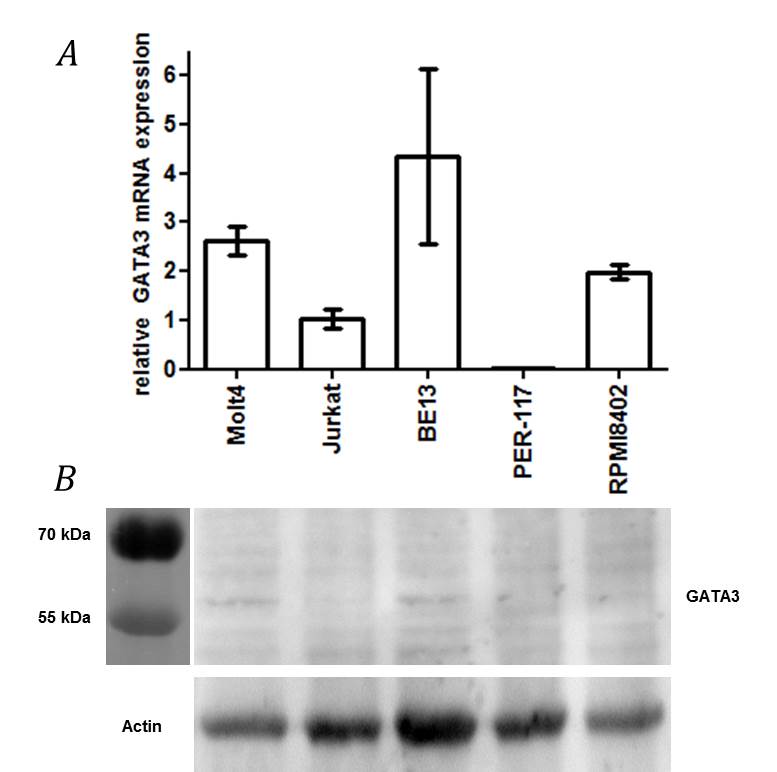

Supplement: Additional file 4: Figure S3. — GATA3 expression in human leukemia cell lines. (A) RT-PCR-based analysis of GATA3 mRNA expression relative to Jurkat indicates differential GATA3 mRNA expression. (B) Western blot analysis revealed differential GATA3 protein expression (predicted molecular weight of 47 kDa). (JPG 35 kb) [file 13045_2016_324_MOESM4_ESM.jpg]

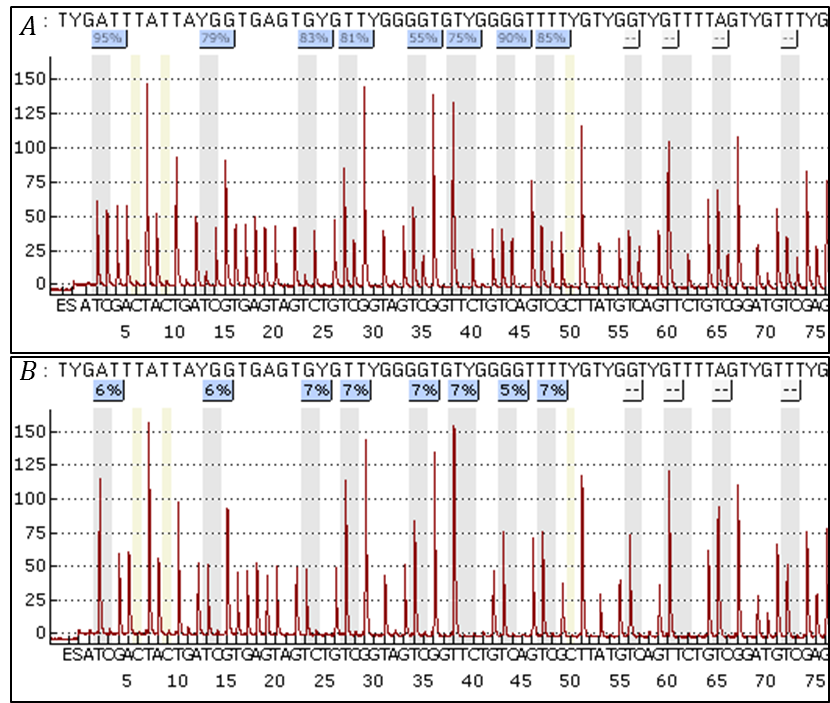

Supplement: Additional file 5: Figure S4. — Pyrograms showing GATA3 DNA methylation in ETP-ALL in eight consecutive CpG sites across 64 bases. The sequence of the GATA3 amplicon is indicated at the top, while the sequence at the bottom of each panel indicates the dispensation order. The grey boxes highlight peaks resulting from sequential dispensations of C and T from which DNA methylation was calculated (blue boxes). (A) Representative ETP-ALL sample exhibiting high GATA3 DNA methylation. (B) Representative ETP-ALL sample exhibiting low GATA3 DNA methylation. (PNG 226 kb) [file 13045_2016_324_MOESM5_ESM.png]

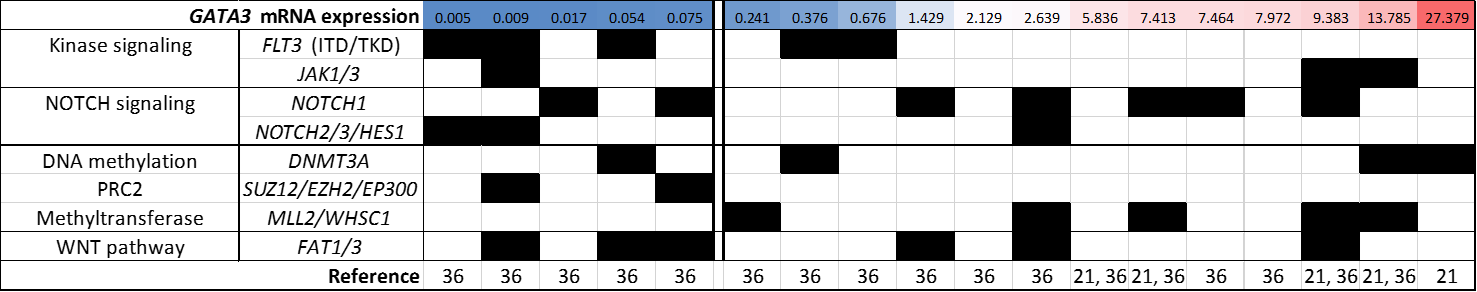

Supplement: Additional file 6: Table S2. — Next generation sequencing comparing the mutational pattern of GATA3low (n = 5) and GATA3high ETP-ALL (n = 13). Sanger Sequencing was performed for selected genes in an extended cohort (n = 70) confirming a high rate of FLT3 mutations in GATA3low ETP-ALL. (PNG 47 kb) [file 13045_2016_324_MOESM6_ESM.png]

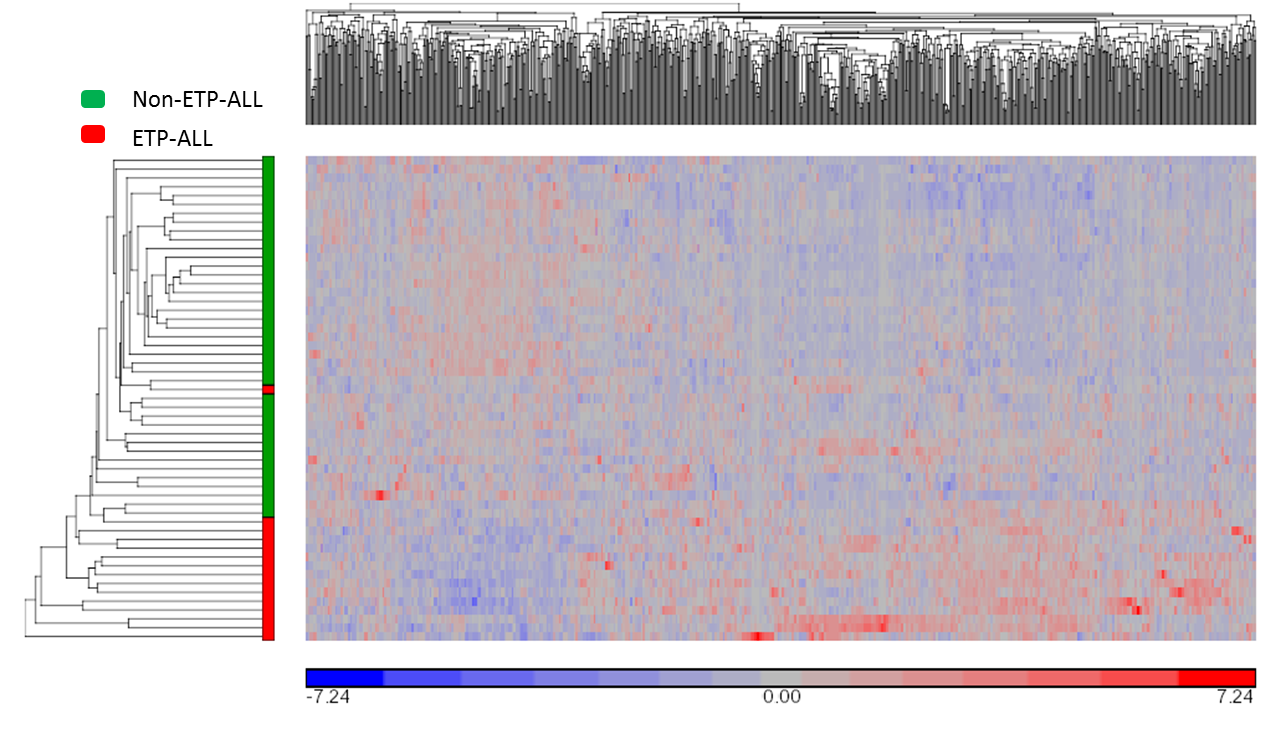

Supplement: Additional file 7: Figure S5. — The GATA3low gene expression profile identified cases with ETP-ALL in an independent cohort of T-ALL. Unsupervised hierarchical cluster analysis of diagnostic bone marrow samples from 55 pediatric patients with T-ALL, using a set of genes defined by low GATA3 expression in adult T-ALL. Classification of ETP-ALL and non-ETP-ALL was performed according to the ETP gene expression signature published along with this dataset. (PNG 484 kb) [file 13045_2016_324_MOESM7_ESM.png]

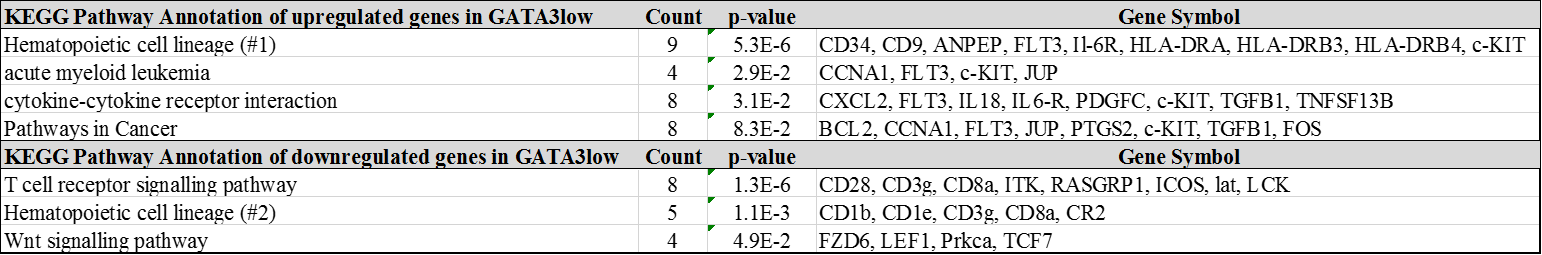

Supplement: Additional file 8: Table S3. — KEGG pathway annotation of DEG in GATA3low versus GATA3high T-ALL patients. Lists of threefold over- and underexpressed genes of GATA3low patients were uploaded to DAVID Bioinformatics Resources v6.7. Listed are selected KEGG annotated pathway elements significantly enriched in GATA3low up- or downregulated DEGs. (PNG 34 kb) [file 13045_2016_324_MOESM8_ESM.png]

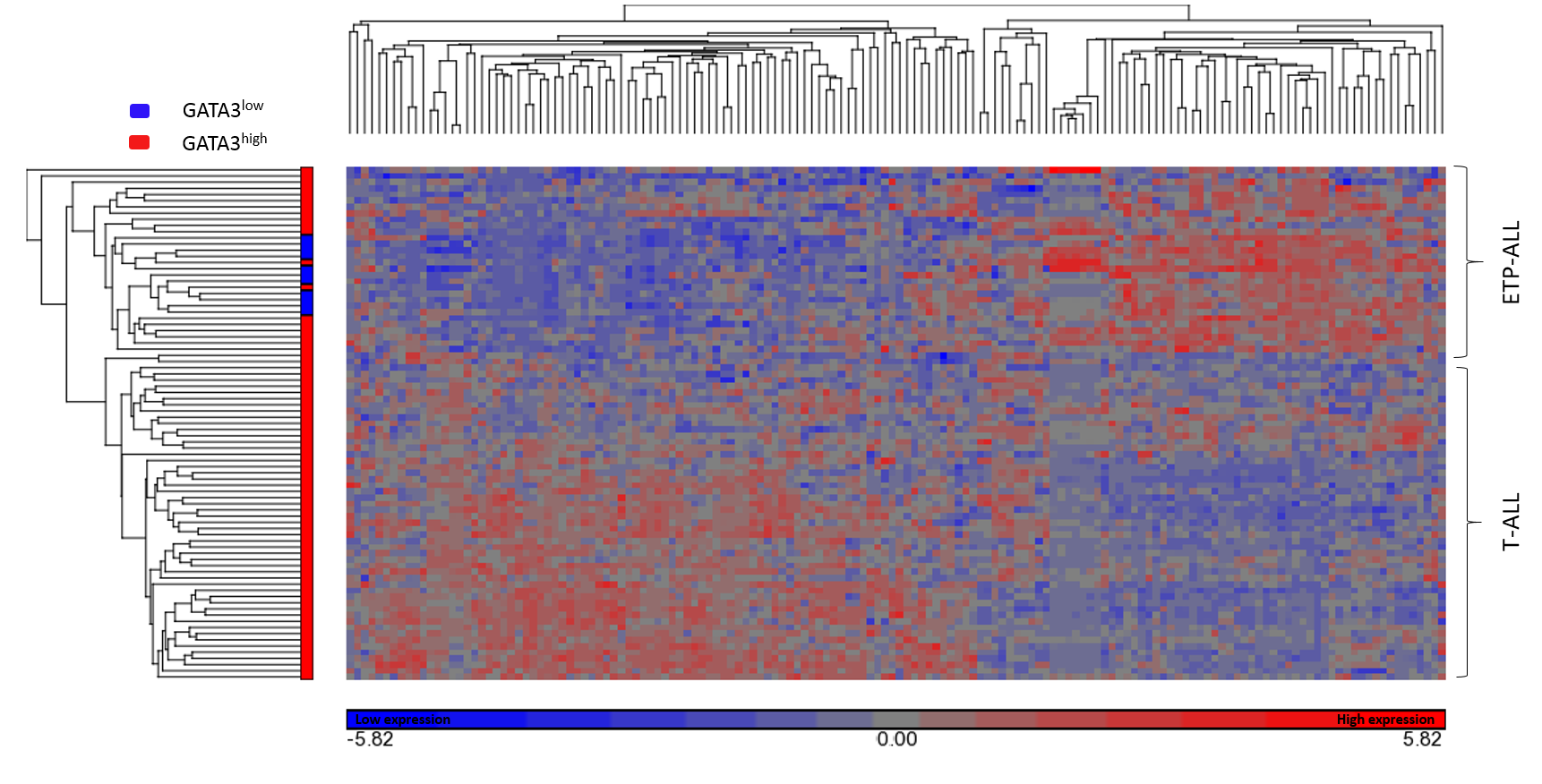

Supplement: Additional file 9: Figure S6. — Validation of the GATA3low ETP-ALL cluster in adult patients with T-ALL using the ETP signature derived from pediatric T-ALL. Unsupervised hierarchical cluster analysis of diagnostic bone marrow samples from 83 adult patients with T-ALL, using a set of genes defined in pediatric T-ALL [20]. Blue = GATA3low ETP-ALL (n = 11), red = GATA3high (n = 72). Patients assigned to ETP-ALL and “typical” T-ALL are indicated. (PNG 748 kb) [file 13045_2016_324_MOESM9_ESM.png]

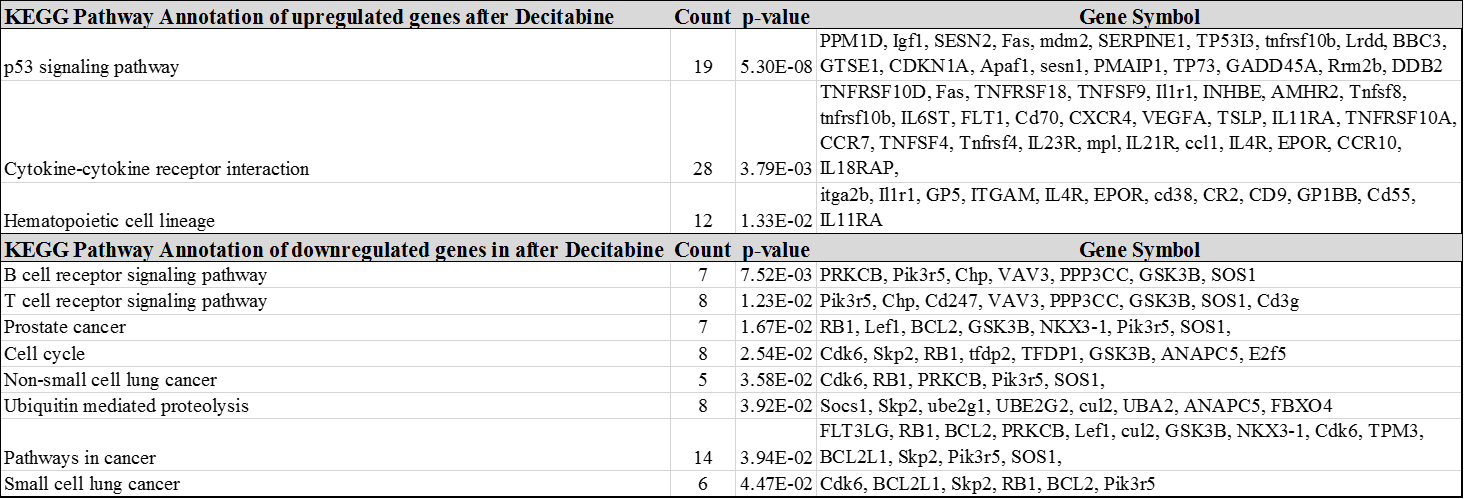

Supplement: Additional file 10: Table S4. — KEGG pathway annotation of DEG in PER-117 cells before versus after treatment with decitabine. Lists of 1.5-fold over- and underexpressed genes of cells treated with decitabine (5 μM) were uploaded to DAVID Bioinformatics Resources v6.7. Listed are selected KEGG annotated pathway elements significantly enriched in cells treated with decitabine. (PNG 61 kb) [file 13045_2016_324_MOESM10_ESM.png]
